# Impediments in foreign collaboration and conducting a high throughput molecular epidemiology research in India, an assessment from a feasibility study

**DOI:** 10.1186/s40064-015-1046-z

**Published:** 2015-06-23

**Authors:** Beenish Iqbal, Idrees Ayoub Shah, Gulzar Ahmad Bhat, Arshid Bashir Bhat, Rumaisa Rafiq, Sumaiya Nabi, Reza Malekhzadeh, Christian C Abnet, Paolo Boffetta, Mazda Jenab, Nazir Ahmad Dar

**Affiliations:** Department of Biochemistry, University of Kashmir, Hazratbal, Srinagar, Jammu and Kashmir India; Digestive Disease Research Center, Shariati Hospital, Tehran University of Medical Sciences, Tehran, Iran; Nutritional Epidemiology Branch, Division of Cancer Epidemiology and Genetics, National Cancer Institute, Rockville, MD 20852 USA; The Tisch Cancer institute and Institute for Transitional Epidemiology, Mount Sinai School of Medicine, New York, USA; Section of Nutrition and Metabolism, International Agency for Research on Cancer (IARC-WHO), Lyon, France

**Keywords:** Feasibility study, Research limitations, Collaboration, ESCC, Kashmir, India

## Abstract

**Background:**

Esophageal cancer is one of the world’s top ten cancers. Its incidence, especially in the form of squamous cell carcinoma, is very high in some Asian regions including Kashmir. Jammu Kashmir and Ladakh are three provinces of Jammu and Kashmir, the northern most state of India. The three regions represent ethnically diverse socio-cultural populations with different incidences of esophageal squamous cell carcinoma (ESCC), a suitable setting for epidemiological studies. Hence, comparing the lifestyle, dietary habits and gene pools between the three regions will help in elucidation of ESCC etiology further. Therefore, to assess the possibility of conducting a larger case control study, we carried out a feasibility study to identify the collaborators as well as to explore patient referral systems and available research facilities in the state.

**Findings:**

We found conducting good cancer molecular epidemiology studies is difficult due to lack of proper research facilities and favourable administrative guidelines. The appropriate storage, transportation and analyses facilities of biological specimens for genome-wide association study and assessment of nutrition and exposure markers are unavailable or not sufficiently developed. Guidelines that can encourage scientific collaborations within the country seem unavailable. However, the administrative guidelines available under which the export of biological specimens out of India for analysis seems impossible. Consequently, Indian researchers are unable to collaborate with foreign scientists and render state of art research facilities inaccessible to them. Scientists in other parts of India may also confront with most of these impediments.

**Conclusion:**

The study found that for conducting conclusive molecular epidemiological studies in India, referral system in hospitals is not systematic, scientific research facilities are inadequate as well as the guidelines for foreign collaboration  are not favourable.

**Electronic supplementary material:**

The online version of this article (doi:10.1186/s40064-015-1046-z) contains supplementary material, which is available to authorized users.

## Background

**Figure 1:**
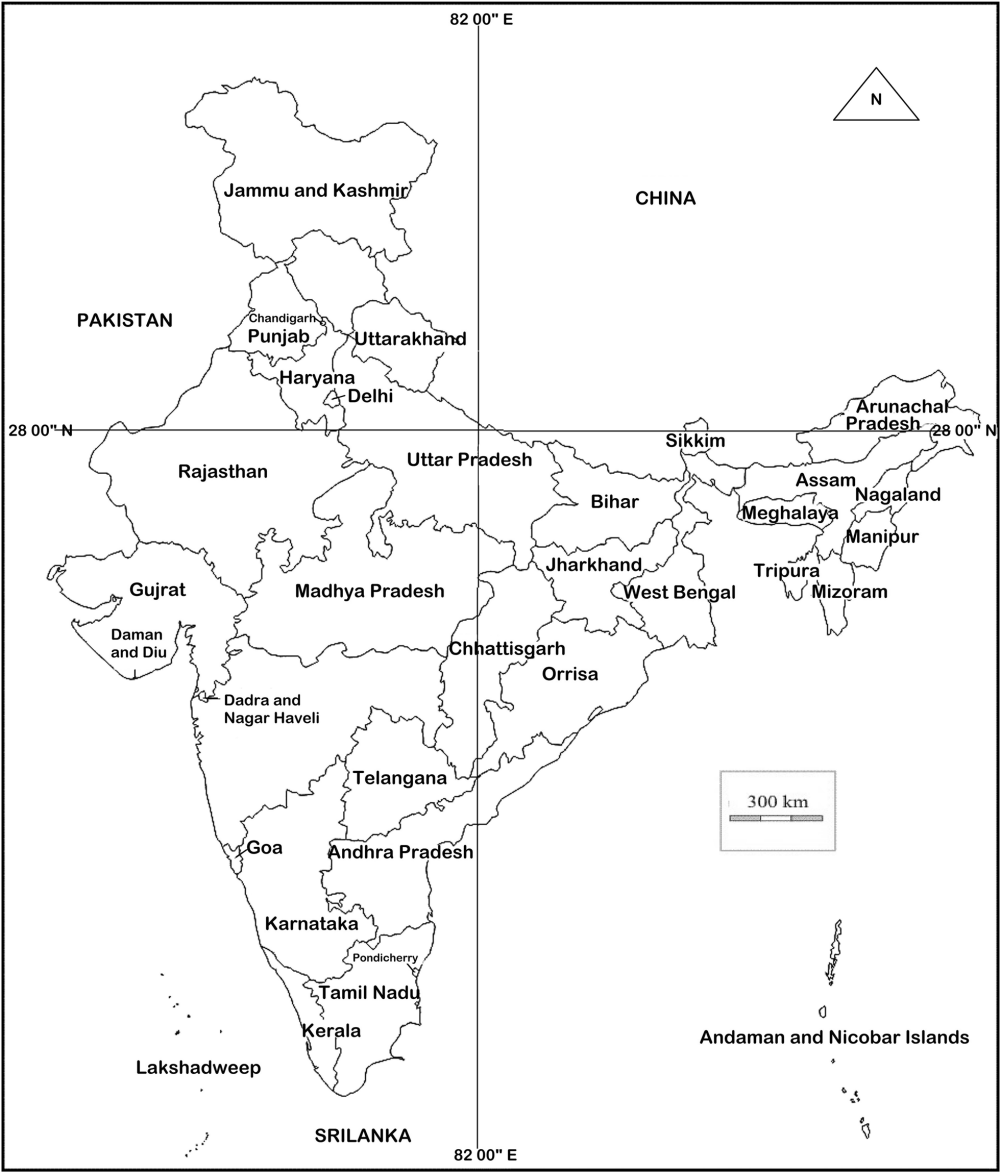
Physical map of India.

Esophageal cancer is the eighth most common malignancy worldwide and the sixth most common cause of cancer deaths (Ferlay et al. 2010). Kashmir Valley, the northern most part of India (Figure 1), lies at the southern edge of “Asian esophageal cancer belt” with a moderately high incidence of esophageal squamous cell carcinoma (ESCC) (Khuroo et al. 1992). Even though several factors have been associated with ESCC risk in the belt including Kashmir (Tran et al. 2005; Dar et al. 2012, 2013a, b; Yu et al. 2010; Kamangar et al. 2009; Nasrollahzadeh et al. 2008), its etiology is still not completely understood. Hence, to gain deeper insight into the etiology of ESCC, more studies on various potential genetic and environmental factors are warranted.

Jammu and Kashmir (J&K) state, consists of three ethnically different regions; Jammu, Kashmir and Ladakh. The inhabitants of these three regions differ in socio-religious backgrounds, life-style, economy and dietary habits and more importantly have a huge differences in ESCC incidence. Jammu and Ladakh regions have very low incidence of ESCC while Kashmir has the highest incidence of ESCC among all the cancers and amongst the three regions (Romero-Gallo et al. 2002; Khuroo et al. 1992). Thus these three heterogeneous populations of J&K provide a suitable setting for conducting epidemiological studies on ESCC. In order to assess the possibility of conducting a big case–control study on ESCC in J&K, a feasibility study (FS) was undertaken to focus on: (1) establishing a collaboration between the clinicians and scientists, (2) building a referral set-up for identification, diagnosis and recruitment of cases, (3) adapting, pre-testing and validation of dietary and lifestyle questionnaires for study populations and (4) assessing the possibility of collection, storage, transportation and analyses of biological specimens for various purposes including genome-wide association study (GWAS).

## Methods

### Referral and speciality set-up and potential for establishing collaborations

The main hospitals from where the cases were recruited are Government Medical Colleges of Jammu and Srinagar and SK-Institute of Medical Sciences (SKIMS), Srinagar which caters the needs from diagnosis to treatment of all cancer patients in the state of J&K. The district hospitals all across J&K refer suspected cancer cases to these hospitals. In Ladakh, no tertiary care hospital is available and cancer patients who visit the existing district hospitals are referred either to Srinagar or Jammu hospitals. To ensure maximum inclusion of incident cases of ESCC and to obtain individually matched controls, all the hospitals were to be brought into the collaborative network to form a base for the referral system linking district hospitals all across the state to speciality hospitals. All the hospitals were contacted for formal collaboration mainly for subject recruitment and referrals. The state lacks private clinics which can provide tertiary care to cancer patients and therefore, were not considered. The study was approved by the institutional ethical committees of SKIMS Srinagar and GMC Jammu.

### Design and pre-testing of questionnaires

The questionnaires [main and food frequency questionnaires (FFQ)] used for Jammu and Ladakh regions were based on, and modified from those developed for two case control studies conducted recently in the two belt regions; Golestan, Iran (Malekshah et al. 2006) and Kashmir (Dar et al. 2012). The comprehensive questionnaires were modified and pre-tested by interviewing patients, as well as attendants and medical staff in the hospitals to evaluate the suitability of the questionnaires for these provinces of the state. For Kashmir, we adopted the questionnaires already designed and tested during our earlier study (Dar et al. 2012). The demographic, socioeconomic and lifestyle related information was obtained by using main questionnaire and FFQ was used to collect information on the consumption of various food items.

### Biological specimen collection, transportation and analysis

It was also important to evaluate the feasibility of collection of quality biological specimens including blood, urine and tissues. The specimen needed to be either processed immediately or transported and stored for further analysis at very low temperatures. The proposed analyses of the biological specimens were for: (a) genetic markers by genome wide association approach, (b) exposure markers/carcinogens like nitrosamines and PAH, (c) expression markers in cancer tissues, and (d) validation of the food frequency questionnaire by measuring nitrogen, potassium, vitamin A and C, carotenoids, fatty acids, etc. It was planned to get most of the analysis done in partner institutions, IARC, France and NCI, USA.

### Validation of FFQ

We preferred to validate the FFQ by assessing the nutritional markers in the body fluids because the measurement errors of the nutritional markers are least in this method. We choose the weekly-method to validate the pretested FFQ. Fifty healthy residents from the different districts were included by administering weekly, the 24 h dietary recall and main FFQ over a period of 6 weeks, besides collecting biological samples viz blood and urine (for details of FFQ validation, please see Additional file 1: Text T1). The 24-h dietary recalls have good quality response rate and do not interfere much with the normal dietary habits of the subjects and were therefore, chosen as the reference method for the FFQ validation (Bohlscheid-Thomas et al. 1997).

## Results

On exploring the possibility of working together with medical centres, we were not able to establish collaboration with most of the medical centres in J&K. The majority of the clinicians particularly in Ladakh region were not ready to participate in the study. We could not find any defined procedure to be followed or specific officials/offices to be approached, to set up a collaboration with hospitals. Even the clinicians who were interested to collaborate were skeptical under such vague situations. However, we could recruit 24/50, 2/10 and 50/100 cases/controls from Jammu, Ladakh and Kashmir, respectively. Subjects with histological confirmation of ESCC were enrolled as cases. There is no state cancer register though hospital cancer registries were available at SKIMS and GMC Jammu only.

Collection of biological specimens was possible from most of the places as the para medical staff, including nurses cooperated and majority of the subjects consented to participate. Storage facilities for biological specimens was not available in the district hospitals except deep freezers (−20°C) in some hospitals which are used for blood storage. However, at SKIMS, GMC Jammu and Srinagar deep freezers (−80°C) are available in a few supporting departments. Transportation of specimens at low temperatures from the place of collection to Kashmir University for storage and processing was very difficult in the existing setup. No proper biobanking facility is available in Kashmir University and even storing the specimen at low temperatures (−80°C) for a longer time is not without a risk due to electricity cuts and lack of backup.

It was foreseen that storage of biological materials will be mirrored and analysed at the International Agency for Research on Cancer (IARC) and partner institutions. However, the analysis of the specimen for genetic, nutritional and exposure markers in foreign laboratories was not possible because of procedural limitations. (For details of the permission process to export samples out of India, please see Additional file 1: Text T2). We explored the possibility of analysing specimen in India also by contacting various laboratories including Dr Lal Path Labs New Delhi and Quest Diagnostics India Pvt. Ltd (QDI). These laboratories could offer specimen analyses for a few nutritional markers only.

## Discussion

We found conducting good cancer molecular epidemiology studies in India is difficult due to lack of proper research facilities and administrative support. Establishing collaboration for subject recruitment (or their referrals) and analyses of biological specimen for genetic, nutritional and exposure markers, in foreign labs, seems difficult.

Establishing collaborations with potential partners was a very difficult exercise. The guidelines and procedures for collaboration are not available. Some clinicians are overburdened which limits their participation in research, while as a section of them lacks preference or motivation for their important role in clinical research. The administration seems unaware of the importance of such studies in the health sector. Without the active participation of the medical fraternity and administration, conducting conclusive epidemiological and other related studies is impossible. Clinicians need motivation and realization of the immense importance of undertaking or participating in public health studies. It remains a challenge for India to develop new models for world-class molecular and environmental epidemiology research involving a strong connection between research institutes and medical centers. Although, there are guidelines from the Medical Council of India which emphasize that quality research and publications be the determinants of career advancements, yet there is a lot more to be done to make such guidelines more effective.

Lack of proper identification and registration of cancer patients in the state is another concern. The existing hospital based cancer registries are incomplete and incoherent in details. This limits the possibility of tracking patients, registering all incident cases without duplication and linking tertiary hospitals with primary and secondary care centres. Hospitals need to be linked, data needs to be shared confidentially and new computer programs and unique registration numbers need to be developed to track and avoid any duplications.

GWAS and assessment of exposure and nutritional markers all need quality bio-specimens, as well as state of the art technology and instrumentation. Limitation of sample storage, transportation at required low temperatures and more importantly lack of infrastructure to analyse them are serious issues. In the absence of these facilities, we could have analysed samples outside India but it was not feasible because of stringent procedural guidelines. Only 10% of samples of a study could be transported outside the country under existing guidelines, which cannot help to reach conclusive findings.

Hence we need to develop state of the art, cutting edge and high through put technology to bring our institutions at par with developed world. We need to make an introspection of our policies and guidelines for analysing the biological specimens in overseas labs. Although we realise that providing more resources and strengthening the national infrastructure for scientific research, can only be solved through time, yet deferring research in the health sector can be detrimental. In view of the immense importance and priority for biomedical research it will be advisable to get benefits of the advanced technology available in other countries.

## Conclusion

It is possible to conduct a large-scale conclusive molecular epidemiology study in Jammu and Kashmir and other regions of India, if policies and guidelines are framed to encourage national and foreign scientific collaboration. Interlinking the hospitals, proper patient registration and referral system on a modern basis should be initiated. Additionally, basic research infrastructure needs improvement and modern scientific laboratories should be established.

## Additional files

Additional file 1:
**Text T1**: Validation of Food Frequency Questionnaire (FFQ). **Text T2**: Procedure to export biological samples.
