# Supplementary material for: Impediments in foreign collaboration and conducting a high throughput molecular epidemiology research in India, an assessment from a feasibility study
Source: Springerplus. 2015 Jun 23;4:287. doi: 10.1186/s40064-015-1046-z (PMC4477010; doi:10.1186/s40064-015-1046-z)
Supplement: Additional file 1: — Text T1: Validation of Food Frequency Questionnaire (FFQ). Text T2: Procedure to export biological samples. [file 40064_2015_1046_MOESM1_ESM.docx]

**Additional File 1:**

**Text T1:** **Validation of Food Frequency Questionnaire (FFQ)**

To validate FFQ we planned to recruit subjects (n = 50) in the identified private clinics in different districts of the valley whom we had to administer the 24 hour dietary recall and the FFQ over a period of 6 weeks, besides collecting the biological samples. From the biological samples we were interested in the analysis of the parameters like; Vitamin A and C; carotenoids - β-carotene, α- carotenoids, Canthaxanthin, β- cryptoxanthin, Lutein, Lycopene, Zeaxanthin, α- tocpherol ,γ- tocopherol; fatty acids; and urinary potassium and nitrogen. The biomarkers like ascorbic acid and carotenoids can either be analyzed from serum or plasma. It was required to note down the time of collection on the blood collection form. The clot could be stored for the extraction of genomic DNA and needed to be stored at -80^0^C. Aliquoting and freezing was necessarily to be completed in 60 minutes. Serum and plasma contain a large number of soluble molecules and most require very low temperature to remain intact (-80^0^C). It has been reported that substantial decrease occurs in the carotenoid levels only if stored at -20^0^C for 6 months. Samples destined for analysis of vitamin C required to be immediately processed, being light sensitive it needed stabilization with meta phosphoric acid, protected from light and rapidly frozen. However, the processing of the samples destined for the analysis of ascorbic acid and carotenoids could be delayed for up to 1 week but the samples must be chilled. Samples destined for other biomarkers like Vitamin A, Vitamin C, Vitamin E, Carotene, and biomarkers of oxidative stress etc. too needed rapid freezing and storage. It is mandatory to protect both blood and serum sample from light. All the samples were to be stored at -80 prior to transportation. 24 hr urine collection is necessary for validation for protein intake. Urine samples were required or determining urinary nitrogen and potassium in grams/ 24 hours and for analysis of biomarkers of exposure to certain carcinogens, e.g. nitrosamines and PAH. Urine samples too needed transportation in frozen state before sending them for the analysis of creatinine, urea nitrogen, sodium, potassium.Almost all the processing protocols as discussed required snap freezing using liquid nitrogen Dewar and transportation in frozen state which was not possible in the available setup

**Text T2: Procedure to export biological samples**

An application in the prescribed format along with requisite documents need to be submitted to International Health Division of ICMR for clearnace to send samples out of country. The documents include a complete research proposal in ICMR format, Material Transfer Agreement, a budget in Indian and Foreign currency, ethical committee clearance, and the mandatory undertaking by the foreign scientist or Lab to support and carry out analyses of the biological samples sent. Once approval is obtained from ICMR, then a No Objection Certificate (NOC) from the Drug Controller General of India (DCGI) is to be obtained for the transportation of the biological samples out of the country. For DCGI to furnish NOC, a mandatory approval from DST (Department of Science and Technology) is needed besides ICMR clearnace. But current regulations permit only that under no circumstances can more than 10% of the biological samples exported. Although retesting of 10% of samples could validate the work of an Indian laboratory, it does not allow for analyses which require measurements on all subjects using technology not currently available in India. Transportation out of country at low temperatures is feasible using commercial contract couriers.
